# *Trichosanthes dioica* root extract induces tumor proliferation and attenuation of antioxidant system in albino mice bearing Ehrlich ascites carcinoma

**DOI:** 10.2478/v10102-011-0028-4

**Published:** 2011-12

**Authors:** Sanjib Bhattacharya, Pallab Kanti Haldar

**Affiliations:** 1 Bengal School of Technology (A College of Pharmacy), Sugandha, Hooghly 712102, West Bengal, India; 2 Department of Pharmaceutical Technology, Jadavpur University, Kolkata 700032, West Bengal, India

**Keywords:** *Trichosanthes dioica*, lipid peroxidation, glutathione, glutathione-*S*-transferase, antioxidative, root

## Abstract

*Trichosanthes dioica* Roxb. (Cucurbitaceae), called pointed gourd in English, is a dioecious climber grown widely in the Indian subcontinent. The present study assessed the influence of treatment of hydroalcoholic extract of *Trichosanthes dioica* root (TDA) on Ehrlich ascites carcinoma (EAC) in Swiss albino mice with effects on antioxidant systems. Twenty-four hours after intraperitoneal inoculation of tumor (EAC) cells in mice, TDA was administered at 25 and 50 mg/kg for 8 consecutive days. On the 9^th^ day, half of the mice were sacrificed for estimation of tumor proliferation, hematological, and hepatic antioxidative parameters. The rest were kept for assessment of survival parameters. TDA exhibited dose dependent and significant increase in tumor weight, tumor volume, packed cell volume and viable cells and reduced non-viable cells and life span of EAC bearing animals. Hematological parameters were significantly worsened in TDA-treated mice. TDA treatment significantly aggravated the hepatic antioxidative parameters. The present study demonstrated that *T. dioica* root possessed tumor promoting activity in EAC bearing albino mice, plausibly mediated by attenuation of endogenous antioxidant systems.

## Introduction

Plants have a long history of use in the treatment of cancer. The approach for minimizing unwanted toxicity is to employ newer natural products that may act by different and distinct mechanism(s) and/or precipitate less serious side effects. A number of plant or other natural products have been studied for anticancer activity leading to the development of several clinically useful anticancer agents (da-Rocha *et al.*, 2001). However, certain plant products demonstrate mutagenic, pro-carcinogenic or tumor promoting effects when administered to normal or tumor bearing animals.

The Ehrlich ascites carcinoma is a transplantable, poorly differentiated, malignant tumor which appeared originally as a spontaneous murine mammary adenocarcinoma. It grows in both solid and ascitic forms. It is a rapidly growing carcinoma with very aggressive behavior and is able to grow in almost all strains of mice. In ascitic form, it has been used as a transplantable murine tumor model to investigate the tumorigenic and antitumor effects of several natural and synthetic chemical substances showing precise and reproducible results (Chen & Watkins, 1970; Segura *et al.*, 2000).

*Trichosanthes dioica* Roxb. (Cucurbitaceae), called pointed gourd in English, *Potol* in Bengali and *Patola* in Sanskrit, is a dioecious climber found wild throughout the plains of North and North-East India from Punjab to Assam and Tripura states of India. It is also grown and commercially cultivated in India, Pakistan, Bangladesh and Sri Lanka for its fruits, a common culinary vegetable in the Indian subcontinent. In India, all parts of this plant have been traditionally used for various medicinal purposes. According to Ayurveda, the traditional system of Indian medicine, its root is a strong purgative. The root has been traditionally used in India as purgative and as tonic, febrifuge, in treatment of jaundice, anasarca and ascites (Kirtikar & Basu, 1935; Anonymous, 1976; Nadkarni, 1976; Sharma *et al.*, 2002). In our earlier studies, we reported antihelmintic effects of leaf and root, antibacterial and antimitotic activities of the root of *T. dioica*. (Bhattacharya *et al.*, 2009, 2010; Bhattacharya & Haldar, 2010a, 2010b). The present study examined the influence of treatment with hydroalcoholic extract of *T. dioica* root on Ehrlich ascites carcinoma (EAC) growth in Swiss albino mice with effects on antioxidant systems *in vivo*.

## Materials and methods

### Plant material

The mature tuberous roots of *T. dioica* were collected during December 2008 from Majdia, Nadia district, West Bengal, India. The species was identified by Dr. M. S. Mondal, at the Central National Herbarium, Botanical Survey of India, Howrah, West Bengal, India, and a voucher specimen (CNH/I-I/57/2009/Tech.II/493) was deposited at Pharmacognosy Research Laboratory, Bengal School of Technology, Delhi Road, Hooghly 712102, India. Just after collection, the plant material was washed thoroughly with running tap water, shade dried at room temperature (24–26 °C) and ground mechanically into a coarse powder.

### Drugs and chemicals

Bovine serum albumin from Sigma Chemical Co., St. Louis, Mo, USA; trichloroacetic acid (TCA) and 1-dichloro-2,4-dinitrobenezene (CDNB) from Merck Ltd., Mumbai, India; thiobarbituric acid (TBA), nitroblue tetrazolium chloride (NBT) from Loba Chemie, Mumbai, India; 5,5’-dithio *bis*-2-nitro benzoic acid (DTNB), phenazonium methosulphate (PMS), reduced nicotinamide adenine dinucleotide (NADH) and reduced glutathione (GSH) from SISCO Research Laboratory, Mumbai, India. All the other reagents used were of analytical reagent grade obtained commercially. Doubled distilled water from all-glass still was employed throughout the study.

### Preparation of extract (TDA)

The powdered plant material (644 g) was macerated at room temperature (24–26°C) with 20% ethanol water (950 mL) for 4 days with occasional shaking, followed by re-maceration with the same solvent for 3 days. The macerates were combined, filtered and evaporated to dryness *in vacuo* (at 35 °C and 0.8 MPa) in a Buchi evaporator, R-114. The dry extract (TDA, yield: 12.15%) was kept in a vacuum dessicator until use.

### Standardization of TDA

TDA was subjected to phytochemical and planar chromatographic studies. Qualitative phytochemical analysis revealed the presence of reducing sugars, amino acids, triterpenoids and steroids in TDA (Harborne, 1998). Presence of cucurbitacin type triterpenoid aglycones in TDA was ascertained by planar chromatography on silica gel pre-coated high performance thin layer chromatography (HPTLC) plates (Silica gel 60 F_254_ Merck, Germany) detected with vanillin-phosphoric acid reagent (Wagner and Bladt, 1996). TDA was dispersed in isotonic saline as per required concentrations and sonicated for 10 min immediately prior to administration.

### Experimental animals

Adult male Swiss albino mice of about 2 months of age weighing 20±2 g were obtained from Laboratory Animal Centre, Department of Pharmaceutical Technology, Jadavpur University, Kolkata, India. The mice were grouped and housed in polyacrylic cages (38×23×10 cm) with not more than four animals per cage and maintained under standard laboratory conditions (temperature 25±2 °C with dark/light cycle 12/12 h). They were allowed free access to standard dry pellet diet (Hindustan Lever, Kolkata, India) and water *ad* *libitum*. The mice were acclimatized to laboratory conditions for 10 days before commencement of the experiment. All procedures described were thoroughly reviewed and approved by the University Animal Ethical Committee, Jadavpur University (Reg. no. 367001/C/CPCSEA).

### Preparation of tumor cells

The transplantable murine tumor cell line namely Ehrlich ascites carcinoma (EAC) cells were obtained from Chittaranjan National Cancer Institute (CNCI), Kolkata, India. The EAC cells were maintained in the ascitic form *in vivo* in Swiss mice by means of sequential intraperitoneal transplantation of 2×10^6^ cells/mouse after every 10 days. Ascitic fluid was drawn form the EAC bearing mouse 8 days after transplantation. The freshly drawn fluid was diluted with ice-cold sterile isotonic saline and the tumor cell count was adjusted to 2×10^7^ cells/mL by sterile isotonic saline.

### Acute toxicity study

The acute oral toxicity of TDA in male Swiss albino mice was studied as per OECD guideline 425 (Anonymous, 2008). The LD_50_ value of TDA was determined using the method of maximum likelihood.

### Effect on normal murine peritoneal cells

The naive mice were divided into three groups (*n*=6). The first group received TDA at the dose of 50 mg/kg b.w., p.o. once for a single day and the second group received the same treatment for two consecutive days. The untreated third group served as control. Peritoneal exudate cells were collected after 24-h treatment by repeated intraperitoneal wash with isotonic saline and counted by a hemocytometer in each of the treated group and compared with those of the untreated group (Sur & Ganguly, 1994).

### Experimental design

The animals were divided into four groups (*n*=12). Except the first group, all groups received 0.1 ml of EAC cell suspension (2×10^6^ cells/mouse, i.p.). This was taken as day ‘0’. The first group served as normal saline control (received isotonic saline, 3 mL/kg body weight, i.p.). The second group served as EAC control. After 24 h of tumor inoculation the third and fourth group received TDA at the doses of 25 and 50 mg/kg body weight, p.o., respectively, for 8 consecutive days. Twenty-four hours after the last dose and after 18 h of fasting, blood was collected from six mice of each group, by cardiac puncture for the estimation of hematological parameters and then sacrificed by cervical dislocation for the study of antitumor and hepatic antioxidative parameters. The remaining six mice of each group were kept alive with food and water *ad libitum* to assess the increase in the life span of the tumor bearing mice. The effect of TDA on tumor proliferation and survival time was assessed by observation of tumor volume, tumor weight, packed cell volume, viable and non-viable cell count, mean survival time (MST) and percentage decrease in life span (% DLS).

### Determination of body weight

The body weight of mice of each group was measured immediately before and 8 days after the treatments.

### Determination of tumor volume and packed cell volume

The mice were dissected and the ascitic fluid was collected from the peritoneal cavity. The volume of collected ascitic fluid was measured by taking it in a graduated centrifuge tube and the packed cell volume was determined by centrifuging at 1 000×*g* for 5 min.

### Determination of tumor weight

The tumor weight was measured by weighing the mice before and after the collection of the ascitic fluid from the peritoneal cavity.

### Determination of tumor cell count

The ascitic fluid was taken in a WBC pipette and diluted 100 times. Then one drop of the diluted suspension was placed on the Neubauer counting chamber and the numbers of cells in the 64 small squares were counted.

### Determination of viability *in vitro*

The cells were then stained with trypan blue (0.4% in isotonic saline) dye (trypan blue dye exclusion assay). The cells that did not take up the dye were viable and those which took the dye were non-viable. The viable and non-viable cells were counted.

### Determination of survival parameters

The animals were observed for their mortality daily until their death or up to a maximum of 40 days. The mortality was monitored by recording mean survival time (MST) and percentage decrease in life span (% DLS) as per the following formula:

$$\%\text{DLS}=\left[\frac{\text{MST of EAC control group}}{\text{MST of TDA treated group}}-1\right]\times 100$$

*Time denoted by number of days.

### Determination of hematological parameters

Collected blood was used for the estimation of hemoglobin (Hb) content, red blood cell (RBC) count and white blood cell (WBC) count (D'Armour *et al.*, 1965; Wintrobe *et al.*, 1961). Differential count of white blood cells (WBC) was carried out from Leishmen stained blood smears (Dacie & Lewis, 1958).

### Determination of hepatic antioxidative parameters

#### Lipid peroxidation (TBARS)

The levels of thiobarbituric acid reactive substances (TBARS) in the liver tissue were measured as reported (Ohkawa *et al.*, 1979). The levels of lipid peroxides (TBARS) were expressed as µmoles of malondialdehyde (MDA)/g of liver tissue.

#### Reduced glutathione (GSH) and glutathione-S-transferase (GST)

The GSH level of liver tissue was determined as per reported method and expressed as µg/g of liver tissue (Ellman, 1959). The enzymatic activity of glutathione-*S*-transferase (GST) was measured by the reported method and expressed as nanomole of CDNB-GSH conjugate formed/min/mg protein (Habig *et al.*, 1974). Total protein of liver was estimated for this purpose by the method of Lowry *et al.* (1951).

#### Superoxide dismutase (SOD) and catalase (CAT)

The activity of SOD in liver tissue was assayed according to the method of Kakkar *et al.* (1984) and the SOD activity was expressed as unit of SOD/mg of liver tissue. Catalase activity was assayed according to the method of Sinha *et al.* (1972). The specific activity of catalase (CAT) was expressed in terms of µmol of hydrogen peroxide decomposed/min/mg of liver tissue.

### Statistical analysis

All data are presented as the mean ± standard error of mean (SEM). The results were analyzed for statistical significance by one-way analysis of variance (ANOVA) followed by Dunnett's *post hoc* test of significance. *p-*values less than 0.05 (*p*≤0.05) were considered statistically significant.

## Results

### Acute toxicity

The oral LD_50_ value of the hydroalcoholic extract of *T.dioica* root (TDA) in mice was 2 800 mg/kg body weight.

### Normal peritoneal cell count

The average peritoneal exudate cell count in naïve mice was found to be 5.41 ± 0.8 × 10^6^. Single treatment with TDA reduced peritoneal cells to 3.73 ± 1.3 × 10^6^, while two-day consecutive treatment reduced the count to 2.67 ± 0.8 × 10^6^. Both reductions were found to be significant (*p*<0.001).

### Body weight

The body weight of mice from EAC control group (after 8 days) was significantly (*p*<0.001) increased when compared with normal control group. TDA significantly (*p*<0.05) increased the body weights of tumor bearing mice in a dose related way as compared to EAC control animals (Table 1).

**Table 1:** Influence of TDA on body weight of normal and EAC bearing mice.

Treatment	Initial body weight (g)	Final body weight (g)
Normal saline (3 mL/kg)	21.12±0.78	21.17±1.20
EAC control (2×10^6^cells/mouse)	21.27±0.63	24.74±0.74§^a^
EAC +TDA (25 mg/kg)	20.65±1.16	26.35±0.58*^b^
EAC+TDA (50 mg/kg)	21.54±0.86	27.48±1.12*^b^

Data are expressed as mean±SEM (*n*=6);

§ final body weight of EAC control group *vs.* normal control group;

* final body weight of all treated groups *vs.* EAC control group,

a *p<*0.001,

b *p<*0.05.

### Tumor proliferation and survival parameters

TDA at 25 and 50 mg/kg body weight increased the tumor volume, tumor weight, packed cell volume and viable tumor cell count significantly (*p*<0.001) in a dose-dependent manner compared to EAC control. Furthermore, TDA decreased non-viable tumor cell counts and increased viable tumor cell counts significantly (*p*<0.001) compared with the EAC control. In the EAC control group, the mean survival time (MST) was 21.42 ± 1.58 days, whereas in TDA treated groups the values were 17.93 ± 1.35 (25 mg/kg) and 12.68 ± 1.12 (50 mg/kg) days, respectively (Table 2).

**Table 2:** Influence of TDA on tumor volume, tumor weight, packed cell volume, cell counts, mean survival time (MST) and decrease in life span (% DLS) in EAC bearing mice.

Treatment	Tumor Volume (mL)	Tumor Weight (g)	Packed cell Volume (mL)	Viable cell count (×10^6^ cells /mL)	Non-viable cell count (×10^6^ cells /mL)	MST (days)	Decrease in life span (% DLS)
Normal saline (3 mL/kg)	–	–	–	–	–	All animals alive	All animals alive
EAC control (2 ×10^6^cells/mouse)	3.08±0.35	3.78±0.87	1.85±0.18	6.13±0.13	0.67±0.04	21.42±1.58	–
EAC+TDA (25 mg/kg)	4.89±0.07*^a^	5.46±0.05*^a^	2.57±0.06*^a^	7.61±0.11*^a^	0.38±0.09*^a^	17.93±1.35*^a^	19.46*^a^
EAC+TDA (50 mg/kg)	6.28±0.27*^a^	6.91±0.26*^a^	3.18±0.21*^a^	9.22±1.07*^a^	0.24±0.03*^a^	12.68±1.12*^a^	68.92*^a^

Data are expressed as mean±SEM (*n*=6);

* all treated groups *vs.* EAC control group,

a *p<*0.001.

### Hematological parameters

Hematological parameters of tumor bearing mice were found to be significantly altered compared to those of the normal saline control group. The leukocyte (WBC) count was found to be increased and RBC and hemoglobin decreased in EAC control animals significantly (*p*<0.001) when compared with the normal control group. Treatment with TDA at both test doses significantly (*p*<0.001) depleted the hemoglobin content and RBC count and elevated the WBC count when compared to the EAC control group animals. In differential count leukocytes, lymphocytes and monocytes were found to be decreased and neutrophils were increased in the EAC control group compared with the normal saline treated group. TDA treatment significantly (*p*<0.001) aggravated these conditions (Table 3).

**Table 3:** Influence of TDA on hematological parameters of normal and EAC bearing mice.

				Differential count		
				
Treatment	Hb content (g/dL)	RBC (cells ×10^6^/mm^3^)	WBC (cells ×10^3^/mm^3^)	Monocytes (%)	Neutrophils (%)	Lymphocytes (%)
Normal saline (3 mL/kg)	12.33±0.15	5.34±0.31	3.36±0.26	1.91±0.06	20.78±1.16	72.46±1.88
EAC control (2 ×10^6^cells/mouse)	6.80±0.46§^a^	3.54±0.17§^a^	4.26±0.72§^a^	1.54±0.05§^a^	58.17±0.98§^a^	46.28±1.57§^a^
EAC+TDA (25 mg/kg)	4.57±0.18*^a^	3.06±0.42*^b^	4.88±0.63*^b^	1.31±0.04*^b^	67.72±1.27*^a^	33.27±2.08*^a^
EAC+TDA (50 mg/kg)	3.46±0.23*^a^	2.68±0.15*^a^	5.71±0.12*^a^	1.12±0.06*^a^	75.46±1.15*^a^	25.29±1.96*^a^

Data are expressed as mean±SEM (*n*=6);

§ EAC control group *vs.* normal control group;

* all treated groups *vs.* EAC control group,

a *p<*0.001,

b *p<*0.05.

### Hepatic antioxidative parameters

#### Lipid peroxidation (TBARS)

The levels of TBARS represented as MDA were significantly (*p*<0.001) increased in EAC control animals when compared to the normal control group. Treatment with TDA dose dependently and significantly (*p*<0.001) elevated the MDA level in tumor bearing mice compared with EAC control animals (Table 4).

**Table 4:** Influence of TDA on hepatic antioxidative parameters of normal and EAC bearing mice.

Treatment	MDA (µM/g of wet liver tissue)	GSH (µg/g of wet liver tissue)	GST (nM CDNB-GSH conjugate/min/mg of protein	SOD (IU/mg of wet liver tissue)	CAT (µM of H_2_O_2_ decomposed/min/mg of wet liver tissue)
Normal saline (3 mL/kg)	167**±**0.02	29.2±0.23	16.4±0.13	8.7±0.28	38±0.14
EAC control (2 ×10^6^cells/mouse)	394**±**0.03§^a^	13.2±0.46§^a^	8.8±0.09§^a^	6.3±0.63§^a^	15±0.25§^a^
EAC+TDA (25 mg/kg)	449**±**0.06*^a^	8.3±0.19*^a^	7.2±0.21*^b^	4.8±0.44*^b^	12±0.17*^b^
EAC+TDA (50 mg/kg)	482**±**0.03*^a^	6.8±0.33*^a^	5.2±0.17*^a^	3.6±0.34*^a^	7±0.30*^a^

Data are expressed as mean±SEM (*n*=6);

§ EAC control group *vs.* normal control group;

* all treated groups *vs.* EAC control group,

a *p<*0.001,

b *p<*0.05.

#### Reduced glutathione (GSH) and glutathione-S-transferase (GST)

In the EAC control group, GSH content and GST activity were found to be significantly (p<0.001) lowered compared to those of normal control animals. The level of reduced GSH and GST activity were found to be significantly (*p*<0.001) diminished by treatment with TDA in tumor bearing mice as compared with EAC control group (Table 4).

#### Superoxide dismutase (SOD) and catalase (CAT)

There were significant (*p*<0.001) reductions in SOD and CAT activities in EAC control group compared with the normal saline group. Treatment with TDA significantly (*p<*0.001) lowered their activities when compared with the EAC control animals (Table 4).

## Discussion

The results of the present study on EAC bearing mice revealed that TDA at the doses of 25 and 50 mg/kg worsened the pathologic condition of tumor bearing animals. TDA brought about marked proliferation of Ehrlich ascites carcinoma as compared with normally grown transplanted tumor, evidenced by a significant rise in body weight, tumor volume, tumor weight, packed cell volume, viable tumor cell count and decrease in normal peritoneal cell count, non-viable tumor cell count, mean survival time and life span of tumor bearing animals. TDA significantly aggravated the altered hematological parameters. TDA also significantly worsened the hepatic antioxidative parameters, viz. lipid peroxidation, reduced glutathione level (GSH), activities of glutathione-*S*-transferase (GST), superoxide dismutase (SOD) and catalase (CAT) in tumor bearing mice.

The effect of TDA treatment on the peritoneal exudate cell count of naïve mice is an indirect method of evaluating its effect on tumor cell growth. Normally a mouse contains about 5×10^6^ peritoneal cells, 50% of which are macrophages. Generally, antitumor agents increase the normal peritoneal cell count, thereby inhibiting indirectly EAC proliferation (Sur & Ganguly, 1994). TDA treatment was found to decrease the peritoneal exudate cell count of normal mice. These results demonstrated the indirect proliferative effect of TDA on EAC cells, which was probably mediated by downregulation of either macrophage or cytokine systems.

Intraperitoneal inoculation of EAC cells resulted in the appearance of ascitic fluid. The ascitic fluid is essential for tumor growth since it constitutes a direct nutritional source for tumor cells (Gupta *et al.*, 2004). Thus rapid increase in ascitic fluid would meet the nutritional requirements of growing tumor cells. This hypothesis was evident in the present study, since inoculation of EAC cells into mice caused significant increase in the body weight of EAC bearing mice. The ncrease was due to accumulated ascitic fluid volume in the peritoneal cavity. TDA treatment dose dependently increased the body weight of EAC treated mice, indicating enhanced tumor growth.

Cancer is a pathological state involving uncontrolled proliferation of tumor cells. The increase in ascitic volume was accompanied by an increase in total cell count. This is associated with an increase in the peritoneal vascular permeability (Fastaia & Dumont, 1976). Treatment with TDA increased intraperitoneal tumor burden, thereby increasing the tumor volume, tumor weight, packed cell volume and viable tumor cell count. The results of Trypan blue dye exclusion assay demonstrated that the viable cell count increased with a decreased count of non-viable cells by TDA treatment. This implied that of TDA favorably affected the proliferation of tumor cells, leading to multiplication of the TDA treated cells. These results could indicate either a direct tumorigenic effect of TDA on tumor cells or an indirect effect, which may involve macrophage inactivation and vascular permeability enhancement. The results of the present study revealed that TDA significantly decreased the life span of EAC bearing mice, presumably due to augmented tumor progression, indicating its tumorigenic potential in tumor bearing mice.

The general properties of cancer chemotherapeutic agents are myelosuppression and anemia (Price and Greenfield, 1958; Hogland, 1982). The anemia encountered in tumor bearing mice is mainly due to reduction in erythrocytes or hemoglobin content, and this may be caused by iron deficiency or by hemolytic or myelopathic conditions (Gupta *et al.*, 2004b). Results of the present study indicated that TDA dose dependently and significantly reduced the erythrocyte count and hemoglobin content compared to EAC control mice. On the other hand, the WBC count was significantly increased compared with EAC control mice. These parameters revealed that TDA exerted a toxic effect on the hematopoietic system.

The redox state of the cell is known to regulate its growth behavior (Pahl and Baeuerle, 1994). The relationship between the endogenous antioxidant systems and growth of malignant cells is a feature observed in several studies. Neoplastic growth has been found to co-exist with impairment in the endogenous antioxidant status (Oberley, 2002). Low activity of the endogenous antioxidant system was found in cancer patients (Balasubramaniyan *et al.*, 1994; Casado *et al.*, 1995) and in experimental carcinoma cell lines (Yellin *et al.*, 1994; Sharma *et al.*, 1993). Considerable evidence suggests that EAC induces oxidative stress in mice (Baumgartner *et al.*, 1978; Gupta *et al.*, 2004a, b; Haldar *et al.*, 2010). Oxidative stress is caused by a relative overproduction of oxidative free radicals or reactive oxygen species (ROS), resulting in lipid peroxidation and subsequently increased malondialdehyde (MDA) and other TBARS levels, which lead to degradation of cellular macromolecules (Yoshikawa *et al.*, 1983). Malondialdehyde (MDA), the end product of lipid peroxidation, a biomarker of oxidative stress, was reported to be higher in carcinomatous tissue than in non-diseased organs (Yagi, 1991; Neilson *et al.*, 1997). The present study revealed that TBARS levels measured as MDA in the EAC control liver tissues were higher than those in normal saline treated liver tissues. A marked increase in the concentration of TBARS in EAC control mice indicated enhanced lipid peroxidation leading to tissue injury and failure of the endogenous antioxidant defense mechanisms to prevent overproduction of ROS. Treatment with TDA enhanced hepatic lipid peroxidation as revealed by elevation of the MDA level. This implied induction of ROS generation by TDA in tumor bearing mice, revealing its pro-oxidant effect.

Glutathione, the most abundant tripeptide thiol, exists as GSH (reduced form) and GSSG (oxidized form) in cells and participates in diverse biological processes, including detoxification of xenobiotics. Glutathione, a potent inhibitor of the neoplastic process, plays an important role in the endogenous non-enzymatic antioxidant system. In reduced form (GSH), it is found in particularly high concentration in the liver and is known to have a key function in the protective process. It acts primarily as reducing agent and detoxifies hydrogen peroxide (H_2_O_2_) in the presence of the enzyme glutathione peroxidase (Arias & Jakoby, 1976; Meister & Anderson, 1983). Besides its involvement in the detoxification process, GSH probably plays also an important role in lymphocyte function. Depletion in GSH content was found to be associated with impaired immune response and increased risk of malignancy (Gmunder & Droge, 1991). Lowered glutathione content was reported in human cancer cell lines (Yellin *et al.*, 1994; Sharma *et al.*, 1993). The depleted reduced GSH may be due to reduction in its synthesis or to its degradation by oxidative stress in EAC bearing animals. TDA treatment significantly lowered the reduced hepatic glutathione content in tumor bearing mice. The results exhibited that the tumor proliferating activity of TDA was accompanied with impairment of the cellular non-enzymatic antioxidant defense system.

Glutathione-*S*-transferases (GSTs) are a family of multigene and multifunctional dimeric enzymes that catalyze the nucleophilic (conjugation) attack of the thiol moiety of glutathione on the electrophilic center of various carcinogens, mutagens and other xenobiotic compounds (Devi *et al.*, 2002). GSTs play an important role in initiating detoxification by catalyzing the conjugation of GSH to electrophilic foreign compounds for their elimination from the system (Mulder *et al.*, 1995). In human cancer tissues lower GST activity was reported (Yellin *et al.*, 1994; Coursin *et al.*, 1996). Our present study revealed a decrease in GST activity in EAC control mice. TDA treatment further diminished hepatic GST activity significantly in EAC bearing mice. Being attenuated by TDA, GSH and GSTs evidently could not act properly to prevent tumor progression in EAC bearing mice.

The enzymatic antioxidant mechanisms are involved in the protection of tissues from oxidative stress by playing an important role in the elimination of oxidative free radicals. Superoxide dismutase (SOD) and catalase (CAT) are involved in the clearance of superoxide and hydrogen peroxide radicals respectively (Oberley, 2002). SOD activity was found to be inhibited in cancer (Jiau-Jian & Larry, 1977; Oberley & Oberley, 1997). Both SOD and CAT activities were reported to be lower as a result of tumor growth (Casado *et al.*, 1995; Marklund *et al.*, 1982). Decreased hepatic SOD and CAT activities were found also in EAC bearing mice (Gupta *et al.*, 2004b; Haldar *et al.*, 2010), and similar findings were observed in our present investigation in EAC control mice. The treatment with TDA, at either of the doses applied further diminished the SOD and CAT activities significantly in a dose dependent manner. Impairment of enzymatic activities, like GSTs, SOD and CAT, in TDA treated tumor bearing mice unveiled attenuation of enzymatic antioxidant defense mechanisms, thus aggravating oxidative stress and resulting in tumor proliferation in mice.

## Conclusion

The present investigation found that 8-day TDA treatment in tumor bearing mice significantly enhanced tumor proliferation and tumor cell viability. It worsened the hematological profile and reduced the survival time (life span), compared with EAC control mice, i.e. normally grown transplanted murine tumor. Further, TDA treatment resulted in significant attenuation of endogenous antioxidant systems by more than one mechanism, involving enhanced lipid peroxidation, impairment of endogenous non-enzymatic (GSH) and enzymatic (GST, SOD, CAT) antioxidant and detoxification systems. From the present investigation it can be therefore concluded that the hydroalcoholic extract of *T. dioica* root induced a remarkable tumor promoting effect on Swiss albino mice bearing Ehrlich ascites carcinoma, mediated plausibly by escalating oxidative stress due to multimodal attenuation of endogenous antioxidant defense mechanisms.
